# Frankincense and myrrh and their bioactive compounds ameliorate the multiple myeloma through regulation of metabolome profiling and JAK/STAT signaling pathway based on U266 cells

**DOI:** 10.1186/s12906-020-2874-0

**Published:** 2020-03-23

**Authors:** Rumeng Gao, Xiaodong Miao, Chengjing Sun, Shulan Su, Yue Zhu, Dawei Qian, Zhen Ouyang, Jinao Duan

**Affiliations:** 1 Jiangsu Collaborative Innovation Center of Chinese Medicinal Resources Industrialization, Jiangsu Key Laboratory for High Technology Research of TCM Formulae, National and Local Collaborative Engineering Center of Chinese Medicinal Resources Industrialization and Formulae Innovative Medicine, Nanjing University of Chinese Medicine, Nanjing, 210023 China; 2 Jiangsu University, Zhenjiang, 212013 China

**Keywords:** Frankincense and myrrh, Multiple myeloma, JAK/STAT signaling pathway, Biomarkers, Metabolomics

## Abstract

**Background:**

Frankincense and myrrh are used as traditional anti-inflammatory and analgesic medicines in China. It has been reported that frankincense and myrrh have significant anti-tumor activities. The present study was designed to investigate the inhibitory efficacy of frankincense ethanol extracts (RXC), myrrh ethanol extracts (MYC), frankincense -myrrh ethanol extracts (YDC), frankincense -myrrh water extracts (YDS) and their main compounds on U266 human multiple myeloma cell line.

**Methods:**

The inhibition effects of cell proliferation was evaluated by MTT assays. Cell culture supernatant was collected for estimation of cytokines. Western blot analysis was designed to investigate the regulatory of JAK/STAT signal pathway. In addition, cell metabolomics based on the ultra-performance liquid chromatography coupled with quadrupole time-of-flight mass spectrometry (UPLC/Q-TOF-MS) had been established to investigate the holistic efficacy of frankincense and myrrh on U266 cells. Acquired data were processed by partial least-squares discriminant analysis (PLS-DA) and orthogonal projection to latent structures squares-discriminant analysis (OPLS-DA) to identify potential biomarkers.

**Results:**

RXC, MYC significantly inhibited the proliferation of U266 cells at dose of 25–400 μg/mL, YDC and YDS at the dose of 12.5–400 *μ*g/mL. 3-O-acetyl-*α*-boswellic acid, 3-acetyl-11 keto-boswellic acid and 11-keto-boswellic acid had the most significant anti- multiple myeloma activities in the 10 compounds investigated, therefore these 3 compounds were selected as representatives for Elisa assay and western blotting experiments. All the extracts and active compounds ameliorated the secretion of cytokines and down-regulated the expression of JAK/STAT signaling pathway-related proteins. Comparing RXC, MYC, YDC and YDS-treated U266 cells with vehicle control (DMSO), 13, 8, 7, 7 distinct metabolites and 2, 2, 3, 0 metabolic target pathways involved in amino acid metabolism, lipid metabolism, vitamin metabolism, arachidonic acid were identified, respectively.

**Conclusions:**

Taken together our results suggest that the frankincense and myrrh and their bioactive compounds inhibit proliferation of U266 multiple myeloma cells by regulating JAK/STAT signaling pathway and cellular metabolic profile.

## Background

Today, multiple myeloma (MM) is the second most common cancer in hematological malignant disease. More than 12 thousand deaths were estimated in 2018, accounted for 2.1% of all cancer deaths and the percent surviving 5 years was 50.7% in 2008–2014, according to epidemiology, surveillance, and end result program (https://seer.cancer.gov/) [1]. The overall efficacy of current conventional therapies for MM, including combined chemotherapy, radiation therapy, stem cell transplantation, new drug treatment, etc., are not ideal. However, the experimental researches on therapeutic effect and mechanism of traditional Chinese medicine on MM have been made a breakthrough.

Frankincense and myrrh, classical traditional Chinese medicine, are commonly used in Chinese medicine as anti-inflammatory and analgesia drugs. Vitro studies have shown that frankincense, which has been commonly believed the efficacy substance is boswellic acid, have anti-inflammatory activity [2], analgesic [3], immunosuppressive and antitumor activities [4]. The main components in myrrh are volatile oils, including monoterpenoid and sesquiterpenoids, having the same effect as frankincense [5–9]. Previous studies have exhibited that the compatibility of frankincense and myrrh have certain impacts against tumors, including Ehrlich ascites [10], prostate cancer cells [11], and breast cancer cells [6], et al. However, there are few studies about the evaluation of the effects on MM and mechanisms.

Owing to the limitation, such as poor repeatability, less quantity of data, and time-consuming of conventional anti-tumor drugs research models, including MTT reduction assay for cellular proliferation, flow cytometry for cellular apoptosis, RT-PCR for gene expression, and Western blot for protein expression [12], the metabolomics has been more and more applied in anti-tumor mechanisms. Metabolomics is one of the newest biology approaches focused on the global metabolic status of the entire organism through non-targeted analysis of metabolites in biological samples and based on the analytical techniques including mass spectroscopy, ^1^H-NMR spectroscopy and liquid chromatography-mass spectroscopy [13]. Notably, cancer is a disease with mitochondrial energy metabolic disorder, which is well recognized as Warburg effect. Therefore, metabolic profiling of cells underlying treatment with anticancer drug candidates is greatly helpful for understanding their action mechanisms.

U266 is a human multiple myeloma cell, and belongs to a suspension cell line, which is the first choice for the study of multiple myeloma diseases in vitro. In this study, we designed to investigate the inhibitory efficacy of Frankincense, Myrrh and their bioactive based on U266 multiple myeloma cells and explain the action mechanism of the anti-multiple myeloma of frankincense and myrrh combining conventional molecular biology with metabolomics.

## Methods

### Chemicals and instruments

U266 human multiple myeloma cells were purchased from the American Type Culture Collection (VA, Catalog No.: CC-Y1527, USA). Fetal bovine serum and RPMI-1640 medium were purchased from Gibco Life Technologies (NY, USA). Methylene blue and dimethyl sulfoxide (DMSO) were purchased from Sigma-Aldrich (MO, USA). IL-6, VEGF ELISA kits were purchased from Nanjing Jiancheng Biotechnology Co., Ltd. (Nanjing, China). JAK1, p-JAK1 STAT3, p-STAT3 and GAPDH antibodies were purchased from Cell Signaling Technology (BSN, USA).

Waters Acquity TM Ultra Performance LC system (Waters, Milford, MA, USA) equipped with a Quattro Micro MS spectrometer and a Waters Xevo TM G2 QTof MS (Waters MS Technologies, Manchester, NH, USA). Deionized water was purified on a Milli-Q system (Millipore, Bedford, MA, USA). Mass Lynx v4.1 workstation was adopted to analyze the data, and Ultra-highspeed centrifuge at low temperature (Thermo Scientific, Waltham, MA, USA); DMI3000M microscope (Leica, München, Germany) were used.

Frankincense (lot number 171010) and myrrh (lot number 171108) were purchased from Suzhou Tianling Chinese Medicine Pieces Co., Ltd. (Suzhou, China). Standards 3-*O*-acetyl--boswellic acid (lot number 20150515), 3*α*-acetyl-20(29)-lupene-24-oic acid (lot number 20150513), *β*-boswellic acid (lot number 20150504), acetyl 11*α*-methoxy-*β*-boswellic acid (lot number 20150519), 3*α*-acetoxy-tirucall-7,24-dien-21-oic acid (lot number 20150523), 11-keto-*β*-boswellic acid (lot number 20150525), 3-acetyl-*β*-boswellic acid (lot number 20150410) ware purchased from Baoji Chenguang Biotechnology Co., Ltd. (Baoji, China) and the purity was greater than 98%. Abietic acid and 2-methoxy-8, 11-epoxygermacra-1(10)-7, 11-trien-6-one were laboratory-made and the purity are greater than 98% determined by UPLC-MS.

### Preparation of drug solution

Ethanol extracts preparation: frankincense, myrrh and frankincense-myrrh pair (1:1 compatibility) were extracted twice with 12 times amount of 95% ethanol, 2 h each time. Combined the two supernatants, then evaporated the solvent to obtain each of the prepared samples. Each sample was separately dissolved in 1% DMSO to prepare 4 mg/mL of the RXC, MYC, and YDC technical liquid concentrates for storage.

Water extract preparation: frankincense-myrrh pair (1:1 compatibility) was extracted twice with 12 times the amount of water, 2 h each time. Combined the two supernatants, then evaporated the solvent to obtain each of the prepared samples. The sample was dissolved in water to prepare 4 mg/mL YDS technical liquid concentrates for storage.

Compounds monomer solution preparation: Each compound monomer was dissolved in 1%DMSO to prepare 1 mmol/L compound monomer technical liquid concentrates for storage.

### Anti-proliferative activity

U266 cells were cultured in RPMI 1640 medium containing 10% FBS and 1% antibiotic-antimycotic and maintained in an incubator at 5% CO2 and 37 °C as ATCC described. A total of 2 × 10^4^ cancer cells in growth media were placed in each well of a 96-well plates. After that, various concentrations of RXC, MYC, YDC, YDS (25, 50, 100, 200 and 400 μg/L) and compounds monomer (5, 10, 25, 50 and 100 μmol/L) were added in each well, then the cells were cultured for 48 h. Each of the drug groups was evaluated in triplicate. After incubation, 10 μL of 0.5 mg/mL MTT solution was added to each well in the dark and cells were incubated for another 4 h. The supernatant was discarded and 150 *μ*L of DMSO was added. Then shaking slightly for 30 min until blue-violet crystals completely dissolved. The absorbance (A) value of each well was measured at 570 nm by a microplate reader and the cell proliferation inhibition rate was calculated. Each experiment was repeated at least three times. Proliferation inhibition rate (%) = (1 - experimental group A value / control group A value) × 100%.

### ELISA assay

IL-6, VEGF kits assay were used to evaluate the change of cytokine. According to the manufacturer’s protocols, 2 × 10^4^ cells were placed in each well of a 96-well plates. After treated with 200, 100, 50 μg/mL RXC, MYC, YDC, YDS and 100, 50, 25 μmol/L BC, AKBA, KBA for 48 h respectively, cell supernatant was collected for Elisa detection.

### Western blot analysis

The myeloma cells of each group were collected after U266 cells were treated with 200 *μ*g/mL of each extracts and 100 *μ*mol/L of monomers for 48 h. The cells were washed with PBS, and extracted with 300 μL RIPA protein extraction lysate for 30 min in an ice bath. The supernatant was collected by centrifuged at 13,000 rpm at 4 °C for 10 min. Protein extracts were separated with SDS-PAGE and transferred to PVDF membrane. After blocking, p-JAK1, JAK1, p-STAT3, STAT3 and GAPDH antibodies were added and incubated. Membranes were washed and incubated in TBST and HRP-conjugated secondary antibody, respectively. The signals were detected using the Bio-Rad ChemiDoc XRS+.

### UPLC/Q-TOF/MS analysis of metabolic Profling

#### Sample preparation for LC-MS

1 × 10^7^ U266 cells were seeded in a 10 cm dish and exposed to 100 μg/mL of RXC, MYC, YDC or YDS or an equal amount of DMSO as a control. Six replicates in separate dishes for each group were analyzed. After 48 h incubation, cells were washed with PBS, then centrifuged and harvested. The cell pellets were rapid quenched with liquid nitrogen, then immediately dissolved in 1.0 mL mixture of methanol/water in ratio of 4:1 (v/v) at − 20 °C and ultra-sonicated for 30 min in an ice bath ultra-sonicator and subsequently centrifuged to collect the supernatant. The supernatant was dried, then resuspended with 100 μL of 80% methanol and filtered through 0.22 mm mesh millipore filters. Drawing 80 *μ*L of supernatant for LC-MS analysis. In parallel a quality control (QC) sample was prepared by mixing the leftover 20 *μ*L of supernatant from each of the 30 samples.

#### UPLC-Q TOF/MS conditions

Chromatographic conditions: Acquity™ UPLC BEH C_18_ column (2.1 mm × 50 mm, 1.7 μm); column temperature: 35 °C; flow rate 0.4 mL/min; injection volume: 3 μL; mobile phase: 0.1% formic acid water (A)-acetonitrile (B). Gradient elution conditions: 0~1 min: 5% B, 1~3 min: 5 ~ 50% B, 3~13 min: 50 ~ 85% B, 13~14 min: 85 ~ 95% B.

Mass spectrometry conditions: samples were ionized using an ESI ion source (ESI^+^/ESI^−^) with a mass scan range of 100 to 1000 m/z; capillary voltage: 3.0 kV; cone voltage: 30 V; extraction voltage of 2.0 V; and ion source temperature of 120 °C; desolvation gas temperature 350 °C; cone flow gas flow 50 L/h; collision energy: 20 ~ 50 eV; desolvation gas flow 600 L/h; activation time: 30 ms; collision gas: high purity nitrogen. A solution of 200 pg/mL leucine- enkephalin (ESI^+^: 556.2771 m/z, ESI^−^: 555.2615 m/z) was used as the locking mass solution to ensure the accuracy during MS analysis.

#### Metabolomics data processing and analysis

The raw data acquired are processed using Mass Lynx software. After chromatographic peak identification, alignment and normalization, a data matrix was obtained: the mass scan range is 100–1000 Da, the retention time range is 0–15 min, the mass deviation threshold is 0.01 Da, the mass window is 0.05 Da, the retention time window is 0.2 min, and the relative peak intensity threshold is 5%, the noise cancellation level is 6.0.

In addition, based on the impurity peaks appearing in the blank solvent control sample, a list of excluded ions was prepared and introduced into the method. The final process will result in a list of data consisting of retention time, m/z values and normalized peak areas. The data was imported into the EZ info 2.0 module for supervised partial least squares discriminant analysis (PLS-DA) and orthogonal partial least squares discriminant analysis (OPLS-DA). The Score-Plot map obtained by OPLS-DA analysis judges and displays the degree of dispersion of the metabolite profiles of samples between groups and extracts potential biomarkers. A point in OPLS-DA represents a variable. The variable importance in the projection (VIP) is measured by the value, and the variable is screened according to the VIP value. The difference between the groups was generally the normal control and the model group. This study directly compared the control group with each drug treated group.

#### Potential biomarkers identification and metabolic pathway analysis

Metabolites with VIP > 1, and those with *P* < 0.05 were compared between the control group compared with each drug treated group as potential biomarkers. They were input into the HMDB (http://www.hmdb.ca), firstly, the molecular weight (deviation 0.5) was selected, the non-endogenous was excluded, and then the relationship with the human cell metabolism was selected, and the unrelated to multiple myeloma was further deleted to obtain the difference metabolite. After the metabolites were identified, information such as the metabolite index number, molecular formula, molecular weight, retention time, VIP value, and relative expression amount were compiled into a table, and introduced into the Metabo database (http://metpa.metabolomics.ca) for metabolic pathway construction.

### Statistical analysis

Data were analyzed using SPSS version 19.0. All values were expressed as the means ± SD of at least three independently performed experiments. Statistical analyses were conducted with Student’s t-test. Differences with *P* < 0.05 were considered to be statistically significant. and *P < 0.05, **P < 0.01, ***P < 0.001 were compared between the treatment group and the control.

## Results

### Determination of chemical components in frankincense-myrrh

Before investigating the anti-tumor activities of frankincense and myrrh, we analyzed the chemical components in the two drugs with the YDC and YDS as representatives. (*n* = 3). Ten components with abundant contents were detected out, and the contents in YDC was much higher in that in YDS (Table 1).

**Table 1:** The contents of 10 components in YDC and YDS

**Components**	**Contents (*μ*g/g)**	
	**YDC**	**YDS**
3-*O*-acetyl-boswellic acid	30.25	_
3α-acetyl-20 (29)-lupene-24-oic acid	36.15	7.23
*β*-boswellic acid	2566.43	51.33
acetyl 11*α*-methoxy-*β*-boswellic acid	118.99	_
3*α*-acetoxy-tirucall-7,24-dien-21-oic acid	701.15	10.98
3-acetyl-11-keto-beta-boswellic acid	2447.87	40.63
11-keto-*β*-boswellic acid	480.12	10.50
3-acetyl-*β*-boswellic acid	765.75	15.30
abietic acid	6.27	_
2-methoxy-8,11-epoxygermacra-1 (10)-7,11-trien-6-one	10,917.79	559.07

### Anti-proliferative activities of frankincense and myrrh on U266 cells

**Fig. 1:**
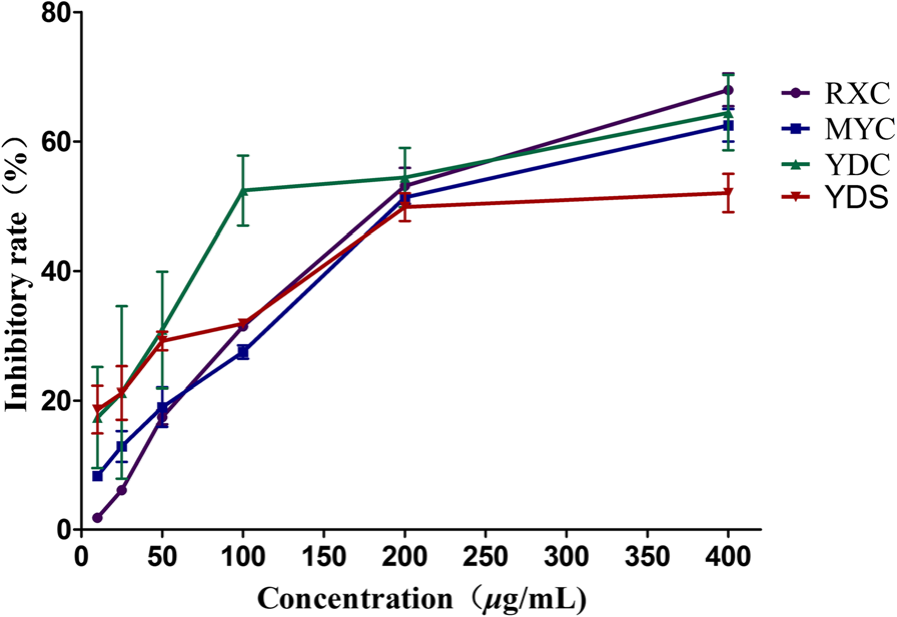
Anti-proliferative effects of RXC, MYC, YDC and YDS on U266 cells. Cell viability of U266 cells analyzed by MTT assay. *P* < 0.05 compared to the control group cells when the concentration is higher than 25 μg/mL

RXC, MYC, at the doses of 25–400 μg/mL, and YDC, YDS, at the doses of 12.5–400 *μ*g/mL, showed dose-dependent anti-proliferative effects on U266 cell growth (Fig. 1). The IC_50_ values of RXC, MYC, YDC, YDS in inhibiting the growth of U266 were 194.81, 229.07, 140.68, 316.75 *μ*g/mL, respectively.

### Evaluation of chemical components bioactivity

Ten components had different degrees of inhibition effects on U266 cells proliferation. Compared with other components, 3-*O*-acetyl-α-boswellic acid (BC), 3-acetyl-11 keto-boswellic acid (AKBA) and 11-keto-boswellic acid (KBA) had the most significant activities, therefore the three components were selected as representatives for Elisa assay and western blotting experiments (Table 2). The IC_50_ values of BC, AKBA, KBA were 35.02, 67.79, 86.56 μmol/L, respectively.

**Table 2:** Inhibition of the proliferation of U266 cells by the 10 chemical components

Components	inhibition ratio (%)					IC50(*μ*mol/L)
	10^−4^ M	5 × 10^−5^ M	2.5 × 10^−5^ M	10^− 5^ M	5 × 10^−6^ M	
3-*O*-acetyl-boswellic acid	84.18 ± 2.38	59.22 ± 6.41	44.06 ± 4.96	11.65 ± 0.09	6.47 ± 2.14	35.02
3*α*-acetyl-20 (29)-lupene-24-oic acid	35.30 ± 0.40	25.11 ± 2.76	17.37 ± 0.88	13.41 ± 1.88	7.54 ± 1.43	293.20
*β*-boswellic acid	43.68 ± 6.75	22.99 ± 3.79	20.18 ± 0.57	16.48 ± 4.43	9.22 ± 0.44	213.36
acetyl 11*α*-methoxy-β-boswellic acid	47.66 ± 3.84	35.18 ± 2.54	31.52 ± 6.38	30.32 ± 7.02	7.45 ± 0.95	103.53
3*α*-acetoxy-tirucall-7,24-dien-21-oic acid	18.90 ± 6.44	8.48 ± 1.66	5.62 ± 0.48	3.91 ± 0.79	1.76 ± 1.44	665.81
3-acetyl-11-keto-*β*-boswellic acid	68.58 ± 0.35	39.13 ± 10.2	13.04 ± 1.42	7.42 ± 3.56	10.14 ± 0.52	67.79
11-keto-*β*-boswellic acid	69.62 ± 1.56	9.05 ± 1.45	7.89 ± 10.91	2.64 ± 0.23	4.17 ± 10.46	86.56
3-acetyl-*β*-boswellic acid	34.27 ± 6.95	26.34 ± 2.65	16.98 ± 6.97	14.24 ± 1.84	5.39 ± 0.81	267.88
abietic acid	24.37 ± 5.41	18.25 ± 4.03	13.70 ± 2.60	7.52 ± 1.27	5.11 ± 2.39	643.48
2-methoxy-8,11-epoxygermacra-1 (10)-7,11-trien-6-one	27.17 ± 3.72	23.42 ± 3.68	22.74 ± 3.71	15.02 ± 0.91	11.09 ± 4.71	1411.47

### Effect of frankincense and myrrh on IL-6 and VEGF secretion of U266 cells

**Fig. 2:**
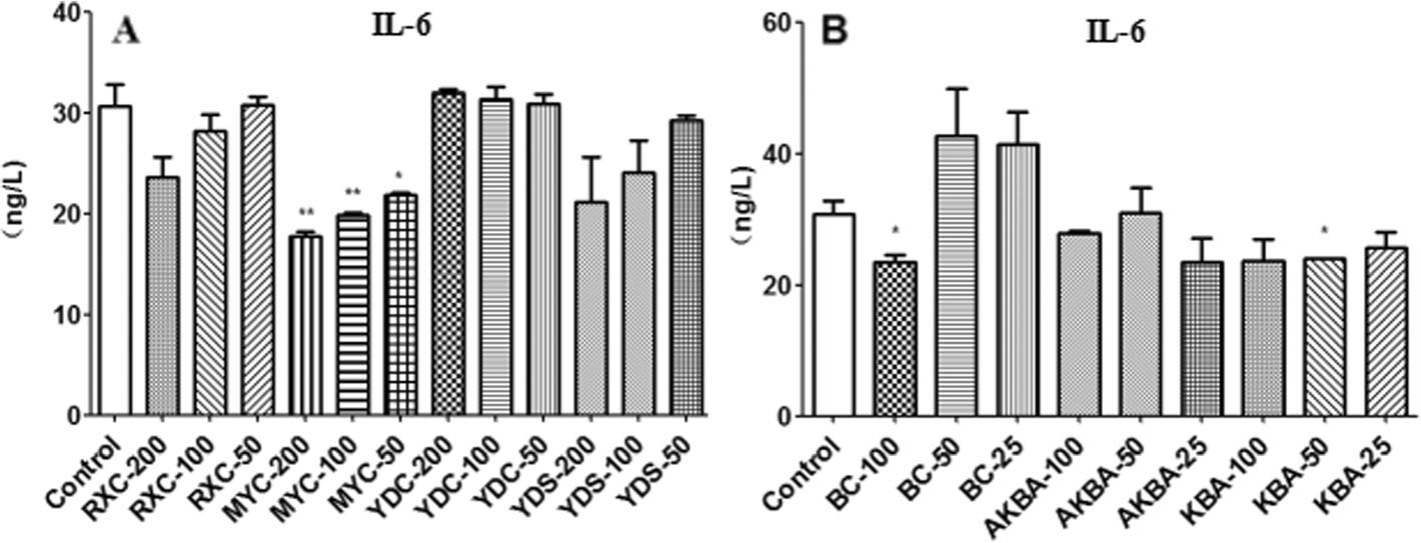
The level of IL-6 secreted by U266 after be treated with extracts or active components. **a** RXC, MYC, YDC, YDS (50, 100 and 200 *μ*g/mL); **b** BC, AKBA, KBA (25, 50 and 100 μmol/L) for 48 h.*P < 0.05, **P < 0.01, vs control group(^−^x ± s, *n* = 3)

**Fig. 3:**
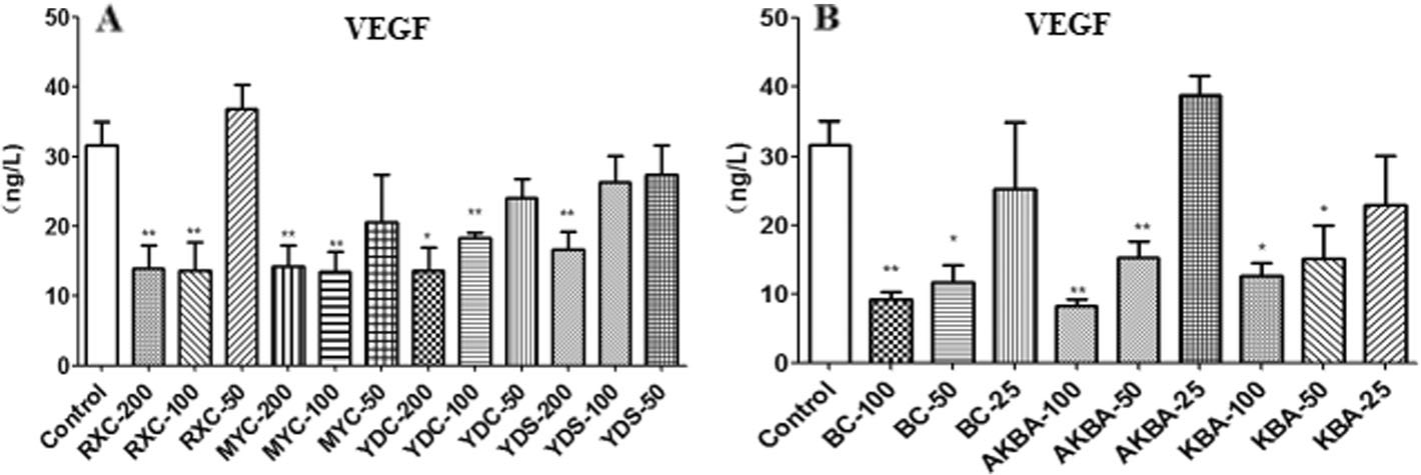
The level of VEGF secreted by U266 after be treated with extracts or active components. **a** RXC, MYC, YDC, YDS (50, 100 and 200 *μ*g/mL); **b** BC, AKBA, KBA (25, 50 and 100 *μ*mol/L) for 48 h.*P < 0.05, **P < 0.01, vs control group(^−^x ± s, n = 3)

A significant reduction in the secretion levels of IL-6 was observed in MYC group compared to the control group, while no significant changes of the secretion levels of IL-6 was observed in other extract groups (Fig. 2a). The results were consistent with the results of BC, AKBA, KBA (Fig. 2b). All the four extracts and active components significantly inhibited the secretion of VEGF in U266 cells (Fig. 3).

### Expression of JAK1, P-JAK1, STAT3 and P-STAT3

**Fig. 4:**
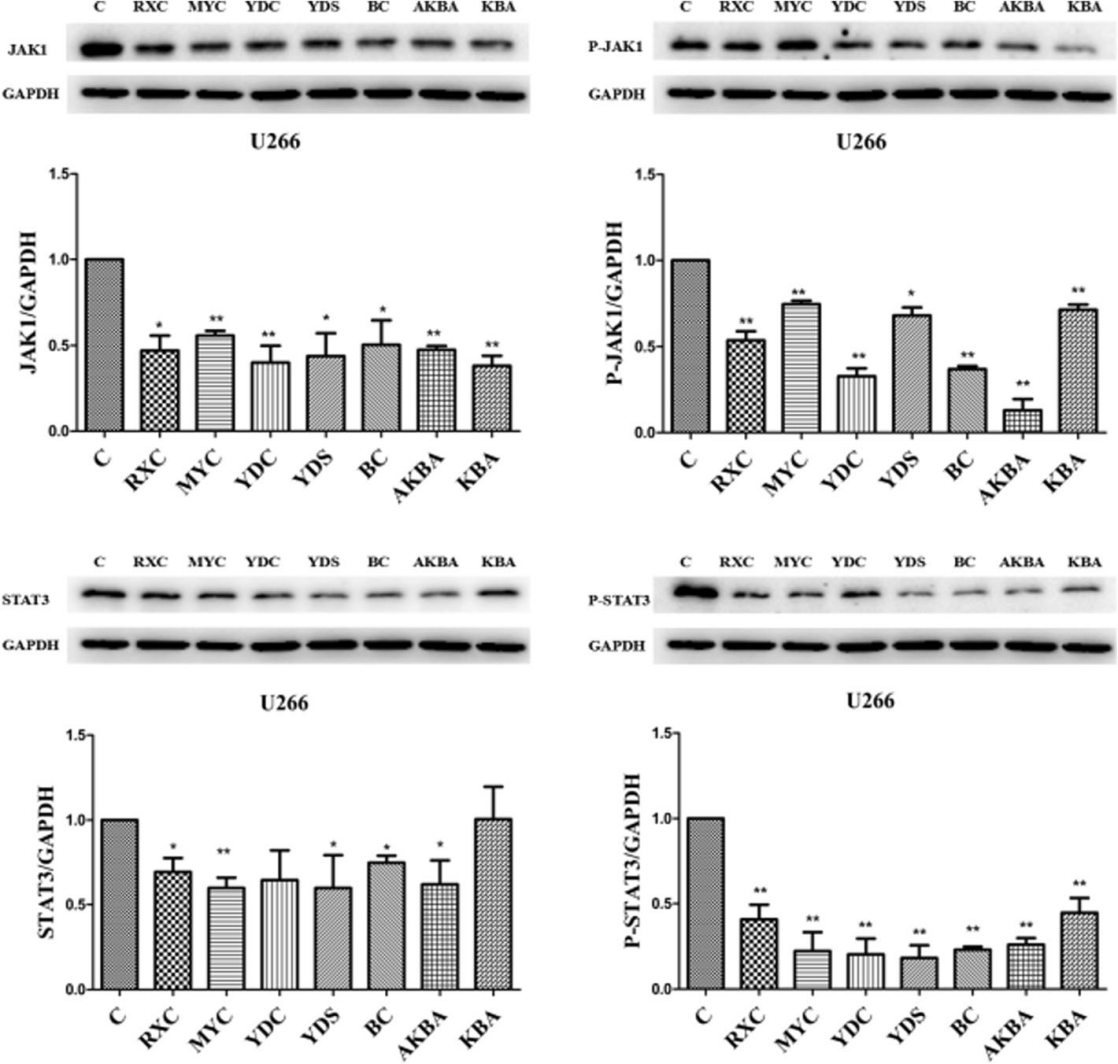
The effect of RXC, MYC, YDC, YDS and BC, AKBA, KBA on JAK/STAT signaling pathway.*P < 0.05, **P < 0.01, vs control group(^−^x ± s, n = 3)

JAK/STAT signal pathway is sustained activation status in control group cells because of the high levels expression of p-JAK1, JAK1, p-STAT3 and STAT3 proteins. Compared with control group, the expression levels of each protein were reduced to varying degrees in and U266 cells after be treated with 200 μg/mL RXC, MYC, YDC, YDS or 100 μmol/L BC, AKBA, KBA. Among them, AKBA had the best inhibitory effect on p-JAK1, KBA had the best therapeutic effect on JAK1, YDS had the most significant effect on p-STAT3, and MYC had a better effect on STAT3 than other treatment groups. Their relative reductions were 0.87, 0.60, 0.82, and 0.40, respectively. (Fig. 4).

### Metabolomics results

#### Metabolomics analysis of RXC, MYC, YDC and YDS-treated cells

**Fig. 5:**
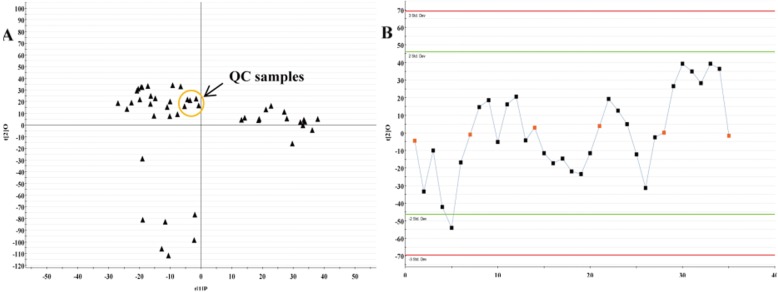
Assessment of QC samples. **a** PCA score plot (PC1 versus PC2) of test samples and QC samples; **b** Trend plot showing the variation of t [1] over all observations. QC samples were colored as red boxes and test samples were colored as black triangle. X axis numbers represented sample number (35 injections); Y axis was arbitrary

As shown in Fig. 5a, the QC samples are tightly clustered together in PCA scores plot, indicating that there was column stability in the whole run. While there had no obvious variation of QC samples over all observations with respect to run order in the trend plot (Fig. 5b). Ten ions chromatographic peaks were selected to evaluated the repeatability of method through six replicates of QC samples. The relative standard deviations (RSD) of ion intensity indicated that the repeatability of method established were well (Table 3).

**Table 3:** Variation condition of ion intensity of selected 10 ions present in the QC samples

T_**R**__m/z pairs	QC1	QC2	QC3	QC4	QC5	QC6	RSD%
1.48_310.1471	15.7756	16.3026	17.3300	17.9054	18.0781	17.7443	5.47
2.96_437.2631	10.9198	11.1992	10.7915	12.1058	10.4506	10.9860	5.08
3.14_525.3208	37.7857	36.6947	36.9523	38.2111	37.0908	36.6130	1.71
5.03_242.1880	41.7784	44.1154	44.9999	45.6316	48.6523	48.6782	5.87
6.92_376.2802	141.8055	146.5531	139.6780	140.8789	135.4626	136.0797	2.91
7.37_194.0911	38.4770	38.8993	39.8244	40.7583	41.4433	42.1215	3.57
8.82_233.1669	96.9642	103.5725	99.2803	92.0731	100.9075	91.6312	4.94
9.88_250.1597	86.5695	87.7531	78.6450	85.6629	83.6178	78.3021	4.88
10.17_311.2141	99.3077	101.6669	102.8994	105.2289	107.2062	107.9275	3.21
13.81_116.9351	365.8650	370.4546	373.0977	373.4281	374.3846	377.9016	1.09

**Fig. 6:**
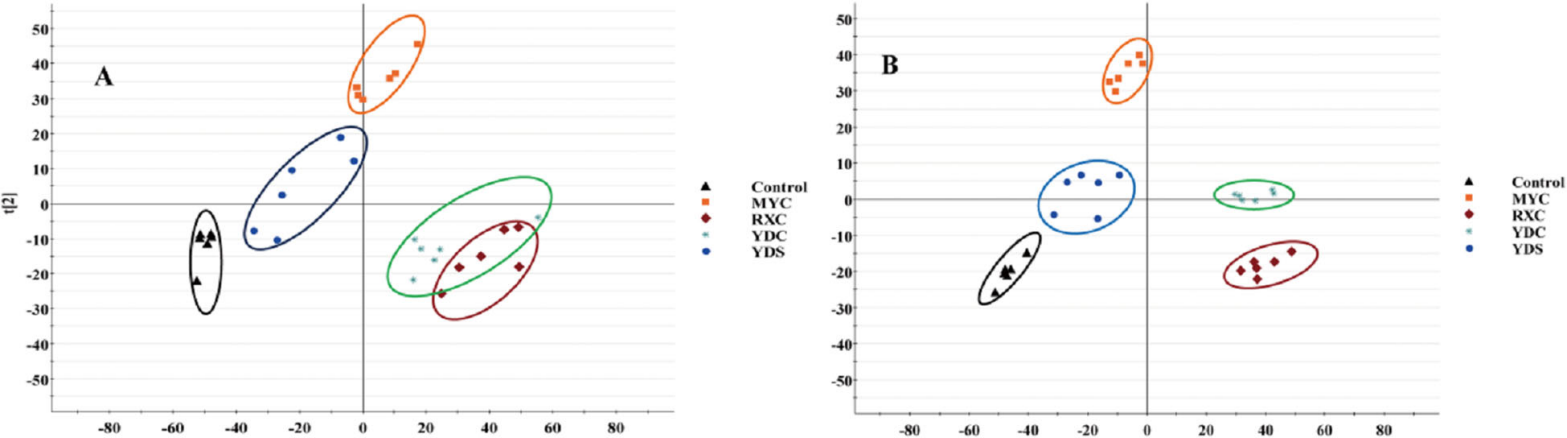
PCA scores plot resulting from UPLC/MS spectra of U266 cells for control and treatment groups. **a** PCA scores plot in positive ion mode. **b** PCA scores plot in negative ion mode

Control and RXC, YDC treated groups were obviously separated along the first principal component, while control and MYC treated groups were significantly separated along the second principal component, and YDS treated group were not significantly different from the control group (Fig. 6).

#### Identification of potential metabolic biomarkers

The differential metabolites were screened according to the S-plot, VIP value, and inter-group peak area t-test between the control group and the treated groups obtained by the UPLC-QTOF/MS analysis platform. These differential metabolites identification were conducted with molecular weight, distribution and polarity of metabolite species by the Human Metabolome Database (http://hmdb.ca/). In this study, thirteen, eight, seven and seven endogenous metabolites involving in multiple metabolic pathways, such as amino acid metabolism, glucose metabolism, lipid metabolism, and vitamin metabolism etc., were ultimately identified which showed significantly changes between control and RXC, MYC, YDC, YDS groups, respectively (P* < 0.05 or P** < 0.01 or P*** < 0.001) (Table 4).

**Table 4:** Potential metabolites selected and identified between treated groups and control group

No.	T_**R**_/min	***m/z***	Identification	Formula	VIP	Trend	HMDB ID
Control vs. RXC							
1	1.12	218.1504	Gamma-glutamyl-L-putrescine	C_9_H_19_N3O_3_	3.24	↓	12,230
2	1.58	120.0872	L-Homoserine	C_4_H_9_NO_3_	3.35	↓	00719
3	2.42	203.0942	3-Hydroxy-N6,N6,N6-trimethyl-L-lysine	C_9_H_21_N_2_O_3_	1.50	↓	01422
4	2.80	180.0756	Hippuric acid	C_9_H_9_NO_3_	2.35	↓	00714
6	3.97	137.0335	Urocanic acid	C_6_H_6_N_2_O_2_	1.14	↑	00301
7	4.64	192.0603	L-Dopachrome	C_9_H_7_NO_4_	5.65	↓	01430
8	5.25	181.0242	Galactitol	C_6_H_14_O_6_	1.51	↓	00107
9	6.52	448.3382	Chenodeoxycholic acid glycine conjugate	C_26_H_43_NO_5_	2.34	↓	00637
10	8.33	315.2742	Pregnenolone	C_21_H_32_O_2_	1.55	↓	00253
11	10.83	524.4035	LysoPC(18:0)	C_26_H_54_NO_7_P	3.56	↑	10,384
12	11.93	305.2663	Arachidonic acid	C_20_H_32_O_2_	1.84	↓	01043
13	13.85	112.9931	Dihydrouracil	C_4_H_6_N_2_O_2_	2.38	↑	00076
Control vs. MYC							
1	1.12	218.1504	Gamma-glutamyl-L-putrescine	C_9_H_19_N_3_O_3_	3.61	↓	12,230
2	1.57	120.0877	L-Homoserine	C_4_H_9_NO_3_	2.62	↓	00719
3	2.43	188.0816	2-Keto-6-acetamidocaproate	C_8_H_13_NO_4_	1.95	↓	12,150
4	2.80	180.0759	2-Methyl-3-hydroxy-5-formylpyridine-4-carboxylate	C_8_H_7_NO_4_	2.06	↓	06954
5	3.62	355.088	5-Amino-6-(5′-phosphoribitylamino)uracil	C_9_H_17_N_4_O_9_P	2.18	↓	03841
6	4.65	176.0805	Indoleacetic acid	C_10_H_9_NO_2_	1.61	↑	00197
7	9.38	496.3721	LysoPC(16:0)	C_24_H_50_NO_7_P	1.96	↑	10,382
8	9.75	149.0322	trans-Cinnamic acid	C_9_H_8_O_2_	2.58	↑	00930
Control vs. YDC							
1	2.80	180.0751	2-Methyl-3-hydroxy-5-formylpyridine-4-carboxylate	C_8_H_7_NO_4_	1.92	↓	06954
2	3.26	160.0514	Aminoadipic acid	C_6_H_11_NO_4_	1.41	↓	00510
3	3.62	355.0865	5-Amino-6-(5′-phosphoribitylamino)uracil	C_9_H_17_N_4_O_9_P	3.71	↓	03841
4	3.96	218.2238	Gamma-glutamyl-L-putrescine	C_9_H_19_N_3_O_3_	2.01	↓	12,230
5	4.00	171.1582	Capric acid	C_10_H_20_O_2_	1.82	↑	00511
6	9.72	149.0310	trans-Cinnamic acid	C_9_H_8_O_2_	1.63	↑	00930
7	10.06	328.2811	Dihydroceramide	C_19_H_39_NO_3_	1.63	↑	06752
Control vs. YDS							
1	1.58	120.0869	L-Homoserine	C_4_H_9_NO_3_	3.42	↓	00719
2	2.43	188.0806	2-Keto-6-acetamidocaproate	C_8_H_13_NO_4_	2.87	↓	12,150
3	2.80	180.0754	2-Methyl-3-hydroxy-5-formylpyridine-4-carboxylate	C_8_H_7_NO_4_	2.43	↓	06954
4	3.96	218.2238	Gamma-glutamyl-L-putrescine	C_9_H_19_N_3_O_3_	2.17	↓	12,230
5	7.47	460.2969	Galactosylsphingosine	C_24_H_47_NO_7_	2.08	↓	00648
6	10.82	524.4034	LysoPC(18:0)	C_26_H_54_NO_7_P	4.53	↑	10,384
7	11.21	289.2684	All-trans-13,14-dihydroretinol	C_20_H_32_O	4.57	↑	11,618

#### Metabolic pathway analysis

**Fig. 7:**
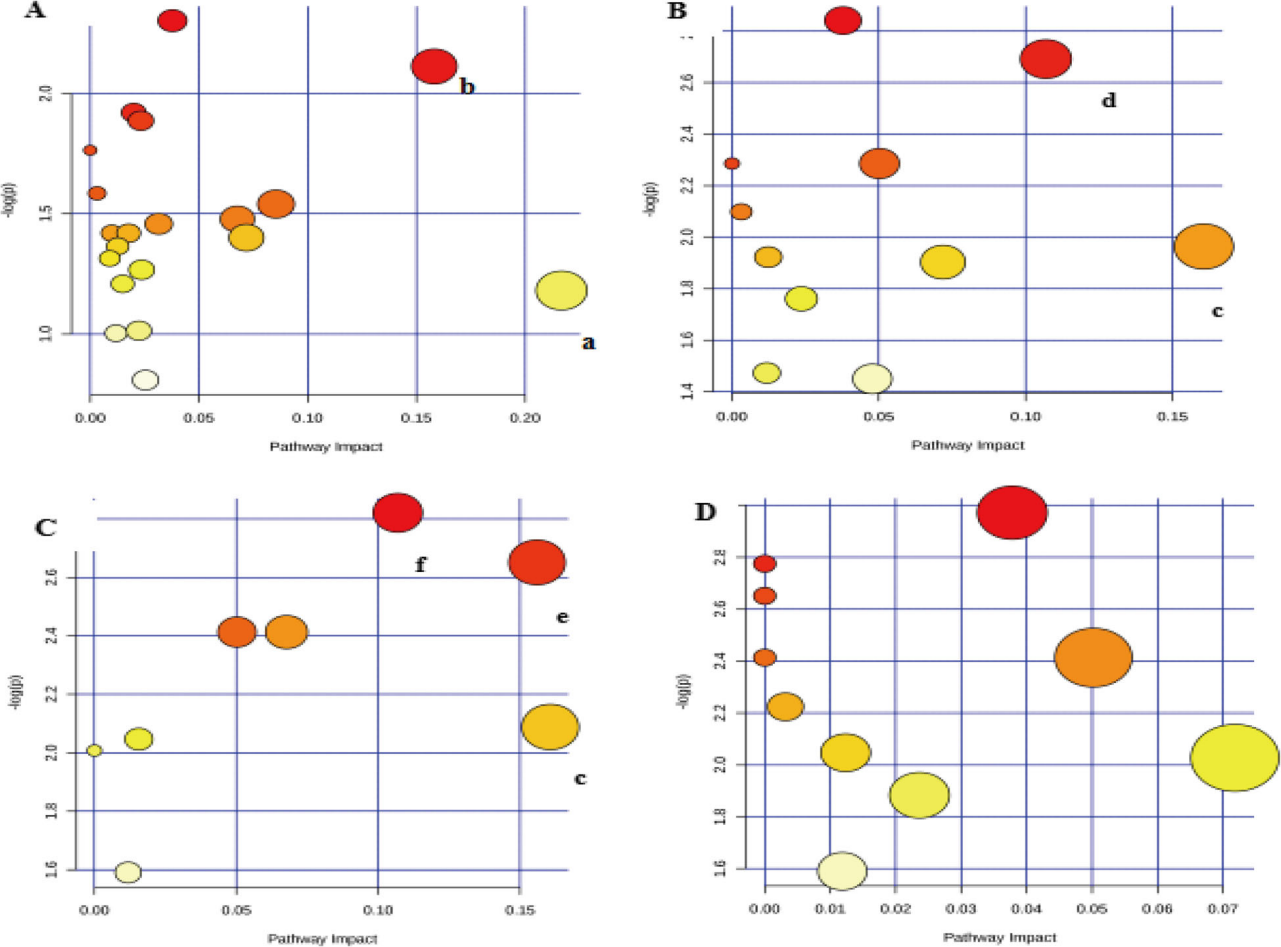
Metabolic pathways constructed by differential metabolites in the RXC, MYC, YDC and YDS group. **a**) - **d** represented RXC, MYC, YDC and YDS groups, respectively. **a** Arachidonic acid metabolism, **b** Retinol metabolism, **c** Phenylalanine metabolism, **d** Riboflavin metabolism, **e** Sphingolipid metabolism

A list of metabolite identifications was imported into the MetPA database, and KEGG database was searched to explore potential metabolic pathways affected by the treatment of RXC, MYC, YDC and YDS. The pathways that with an impact value above 0.10 were screened out as the potential target pathway. RXC group mainly affected the retinol metabolic pathway and the arachidonic acid metabolic pathway (Fig. 7a). MYC group mainly affected the riboflavin metabolic pathway and the phenylalanine metabolic pathway (Fig. 7b). The metabolites affected by YDC group were involved in riboflavin metabolism pathway, sphingolipid metabolism pathway, phenylalanine metabolism pathway (Fig. 7c), however there was no potential target pathway revealed in the YDS group (Fig. 7d).

## Discussion

The aim of the present study was to assess the inhibitory effects of frankincense and myrrh, and try to elucidate the anti- myeloma mechanism through metabolic profiling. In this study, RXC, MYC, YDC and YDS showed dose-dependent anti-proliferative effects on U266 cell growth at the doses of 25–400 μg/mL. BC, AKBA and KBA had the most significant activities, therefore the three components were selected as representatives for Elisa assay and western blotting experiments. Studies have shown that these three components have significant antitumor activity. Park B et al. reported that AKBA concentrations exceeding 50 μmol/L in U266 cells down-regulated CXCR4 expression, which is associated with the invasion and metastasis of cancer cells [14]. Zhao W et al. showed that BC-4 blocked the invasion and metastasis of B16F10 mouse melanoma cells by inducing differentiation and blocking the cell population in G1 phase and inhibiting topoisomerase II activity [15].

MM is a tumor that occurs in the terminal stage of B cell differentiation. Some cytokine abnormalities can affect the activation, development and differentiation of normal B cells, and play a key role in the occurrence and development of MM [16]. IL-6 is the most important cytokine to maintain MM cell survival and promote its proliferation. Tsuyama N et al. confirmed that MM cells expressing IL-6 have higher malignancy, faster proliferation, and are prone to drug resistance [17]. IL-6 regulates the functional status of tumor cells mainly through two signaling pathways, namely, the Ras-dependent MAPK pathway, that may be associated with IL-6 proliferation of MM cells, and the Ras-independent STATs pathway, that may be associated with IL-6 inhibition of apoptosis in MM cells [18]. VEGF is a highly specific pro-vascular endothelial growth factor that promotes increased vascular permeability, extracellular matrix degeneration, vascular endothelial cell migration, proliferation, and angiogenesis [19]. MM cells promote the expression of IL-6 in BMSCs of bone marrow stromal cells by expressing VEGF and its receptors Flt-1 and KDR, thereby promoting the proliferation of MM cells and inhibiting apoptosis [20]. In this study, MYC significant reduced in the secretion levels of IL-6 and all the four extracts and active components significantly inhibited the secretion of VEGF in U266 cells.

We further investigated the expression levels of JAK1, P-JAK1, STAT3 and P-STAT3 to prove the cell apoptosis triggered by RXC, MYC, YDC and YDS. JAK/STAT pathway is one of the most common tumor cells apoptotic signaling pathways involving numerous cytokines, growth factors and hormones [21]. Sustained activation of JAK/STAT signaling is frequently linked to cancer cell proliferation, survival, metastasis, tumor immunosuppression, and angiogenesis, and IL-6-mediated apoptosis inhibitory [22–25]. Kunnumakkara AB et al. evaluated the regulation of AKBA in U266 and MM.1S human multiple myeloma cells, SCC4 human oral squamous cells and A293 cells in vitro. The data showed that AKBA suppresses IL-6-induced STAT3 activation on those cells and phosphorylation of both Jak2 and Src. In addition, the significant inhibition of STAT3 activation by AKBA led to the suppression of gene products involved in proliferation (cyclin D1), survival (Bcl-2, Bcl-xL and Mcl-1), and angiogenesis (VEGF) [26]. Using p-STAT3, the final link of the pathway, as a standard to evaluate the regulation effects of JAK/STAT signaling pathway by treated groups, the results showed there were significant decrease in each treated groups (*P* < 0.01), however, there was no significant difference between the extract groups.

Meanwhile, we investigated the metabolic profile of U266 cells regulated by RXC, MYC, YDC and YDS. Control and RXC, YDC treated groups were obviously separated along the first principal component, that indicated the cellular metabolic profile were significantly altered under the treatment of RXC and YDC. The results were consistent with anti-proliferative activity. The differential metabolites were screened by the UPLC-QTOF/MS analysis platform. In this study, thirteen, eight, seven and seven endogenous metabolites showed significantly changes between control and RXC, MYC, YDC, YDS groups, respectively. Because the metabolites in RXC and YDC treated groups were the most significantly changed, further studies were focused on the metabolites L-homoserine, *γ*-glutamine putrescine and LysoPC (18:0), and arachidonic acid metabolism, retinol metabolism, phenylalanine metabolism, riboflavin metabolism, sphingolipid metabolism and other pathways. Which altered obviously in both RXC and YDC treated groups.

Lyso-PC, derived from glycerophospholipid metabolic pathway, has been confirmed to increase oxidative stress by inducing antibody formation and stimulating macrophages, leading to lipid peroxidation and vascular endothelial dysfunction in inflammatory state [27]. Moreover, LysoPC is an intermediate product of phospholipid choline (PC) metabolism, and PC is the main lipid component of biofilm, so the change of LysoPC reflects the state of cell membrane. Compared with the control group, the content of LysoPC (18:0) was significantly increased in RXC and YDC groups, indicating that RXC and YDC can induce oxidative stress damage and destroy the stability of the cell membrane [28].

*γ*-glutamine putrescine is involved in the degradation pathway of putrescine II, which is formed by putrescine and L-glutamic acid driven by ATP. Putrescine is important for maintaining the basic function and viability of cells. Recent studies have found that excessive accumulation of putrescine in cells can lead to increased uptake of the medium or increased intracellular synthesis, leading to apoptosis [29, 30]. In our study, the levels of γ-glutamine putrescine in RXC and YDC groups were significantly lower than those in the control group, indicating that RXC and YDC may block the degradation pathway of putrescine in U266 cells, and then the accumulation of γ-glutamine putrescine induce apoptosis of tumor cells.

L-homoserine is an intermediate product of methionine biosynthesis, involved in the methionine metabolic pathway. Methionine is an essential amino acid that plays an important role in protein synthesis and other biochemical processes. Studies have shown that methionine can be participate in the methylation of DNA and proteins as a methylation donor, thereby regulating the expression of genes and proteins [31]. In addition, L-methionine can inhibit the proliferation of cancer cells by inhibiting post-translational modification of the gene p53 [32]. In the present study, the L-homoserine in RXC and YDC groups were at low levels, indicating that these two may increase the activity of methionine synthesis-related enzymes, promote the conversion of L-homoserine to methionine, and further cause methylation of U266 cell DNA, thereby inhibiting cell proliferation.

Epoxyeicosatrienoic acids (EETs) are epoxy derivative of arachidonic acid formed by cytochrome P-450 (CYP) cyclooxygenase. Cheranov et al. [33] reported that EETs induced endothelial angiogenesis by stimulating VEGF expression through Src-dependent STAT-3. Research shows 17-ODYA is an effective cycysase inhibitor, which can inhibit the expression of EETs and thus reduce the production of VEGF [34]. So, RXC and YDC may inhibition of EETS secretion on cytochrome P-450 pathway promotes apoptosis of U266 cells.

**Fig. 8:**
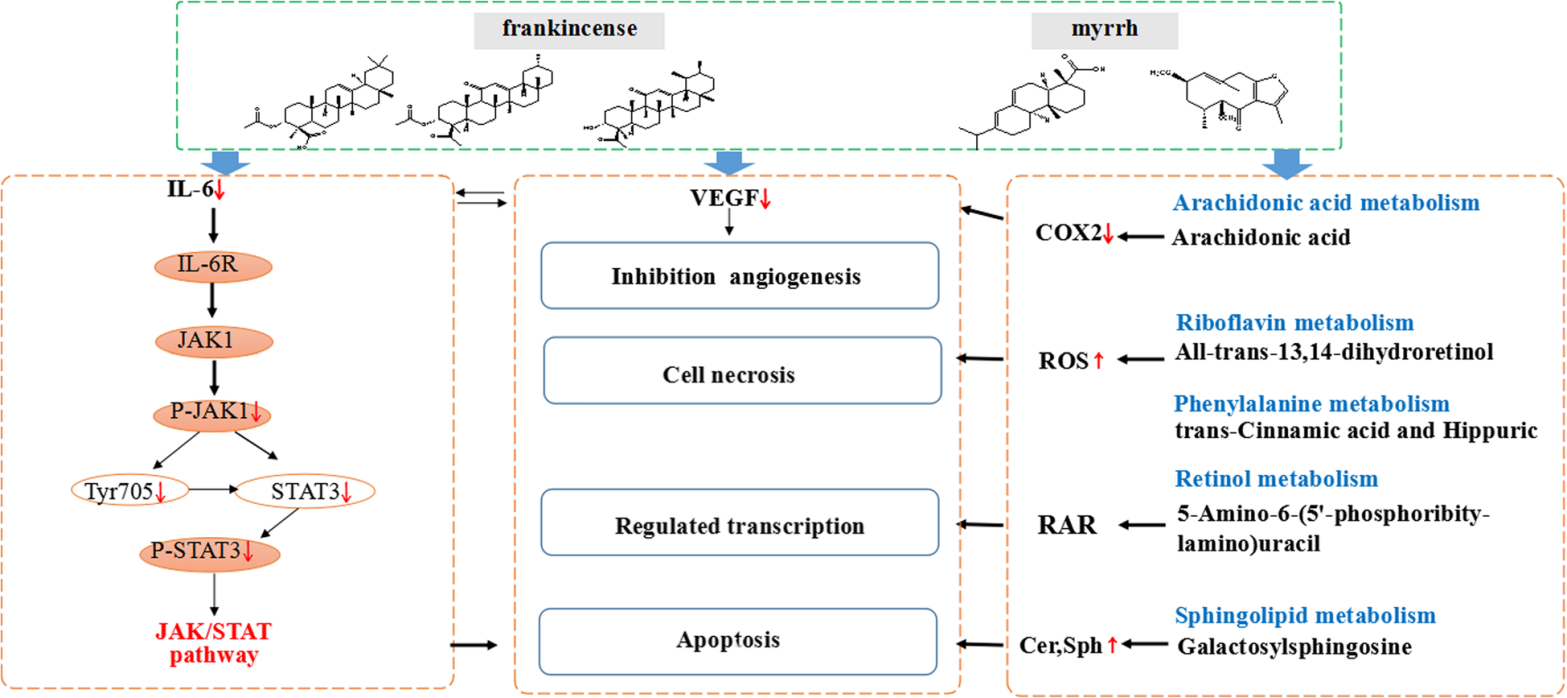
The potential mechanism of frankincense and myrrh against MM

Through the above pharmacodynamic research and preliminary discussion on signal pathway and metabolic pathway, we speculate that frankincense and myrrh first inhibit the secretion of IL-6 in myeloma cells, that may affect the amount of IL-6 in the bone marrow microenvironment. The decrease in the amount of IL-6 causes an activation of JAK/STAT signaling pathway. Meanwhile, the stimulation of frankincense and myrrh result in the changes of metabolic pathway, thereby inhibiting tumor angiogenesis, inducing apoptosis, causing cell necrosis. The potential mechanism of frankincense and myrrh against multiple myeloma is shown in Fig. 8.

## Conclusions

In summary, all of the treatment with frankincense ethanol extracts, myrrh ethanol extracts, frankincense-myrrh ethanol extracts and frankincense-myrrh water extracts showed significant anti-proliferation effect on U266 cells at the dose of 25–400 μg/mL. The inhibition effects on U266 cells proliferation of all extracts and active components were associated with the suppressed activation of JAK/STAT signaling pathways and regulation of the bone marrow microenvironment. In addition, we speculate that the proliferation inhibitory effects of U266 cells may also owe to the regulation of vitamin metabolism, amino acid metabolism and lipid metabolism. This study provides a basis for studying the mechanism of the compatibility of frankincense myrrh from the perspective of endogenous metabolites. However, the specific mechanism of action remains to be determined, and there is a lack of normal control of PBMC cells and validation of metabolites in current studies. We believe that PBMC/ in vivo toxicity and metabolite validation are necessary, and we will conduct validation tests for those in the future.

## Supplementary information

**Additional file 1.Figure S1-S15** Untreated GAPDH, p-JAK1, JAK1, p-STAT3 and STAT3 protein bands images (n=3).

## Data Availability

Data are all contained within the manuscript.

## Abbreviations

AKBA: 3-acetyl-11 keto-boswellic acid
BC: 3-*O*-acetyl-*α*-boswellic acid
IL-6: Interleukin-6
JAK/STAT: Janus kinase/signal transducer and activator of transcription
KBA: 11-keto-boswellic acid
MM: multiple myeloma
MTT: 3-(4,5-dimethylthizaol-2-yl)-2,5-diphenyl tetrazolium bromide
MYC: Myrrh ethanol extracts
RXC: Frankincense ethanol extracts
VEGF: Vascular endothelial growth factor
YDC: Frankincense-myrrh ethanol extracts
YDS: Frankincense-myrrh water extracts
