# Supplementary figures and images for: Frankincense and myrrh and their bioactive compounds ameliorate the multiple myeloma through regulation of metabolome profiling and JAK/STAT signaling pathway based on U266 cells
Source: BMC Complement Med Ther. 2020 Mar 23;20:96. doi: 10.1186/s12906-020-2874-0 (PMC7092432; doi:10.1186/s12906-020-2874-0)

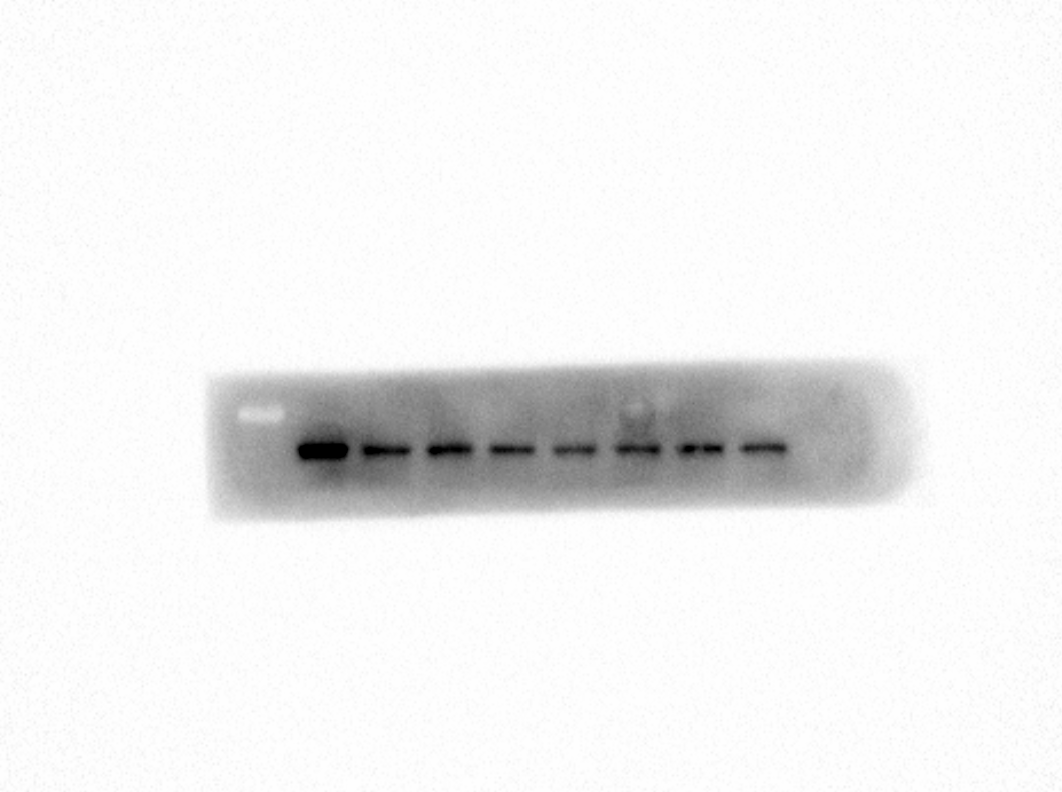

Supplement: Supplementary file 1 — Additional file 1.Figure S1-S15 Untreated GAPDH, p-JAK1, JAK1, p-STAT3 and STAT3 protein bands images (n=3). [file 12906_2020_2874_MOESM1_ESM.zip › 12906_2020_2874_MOESM1_ESM/Figure S1.tif]

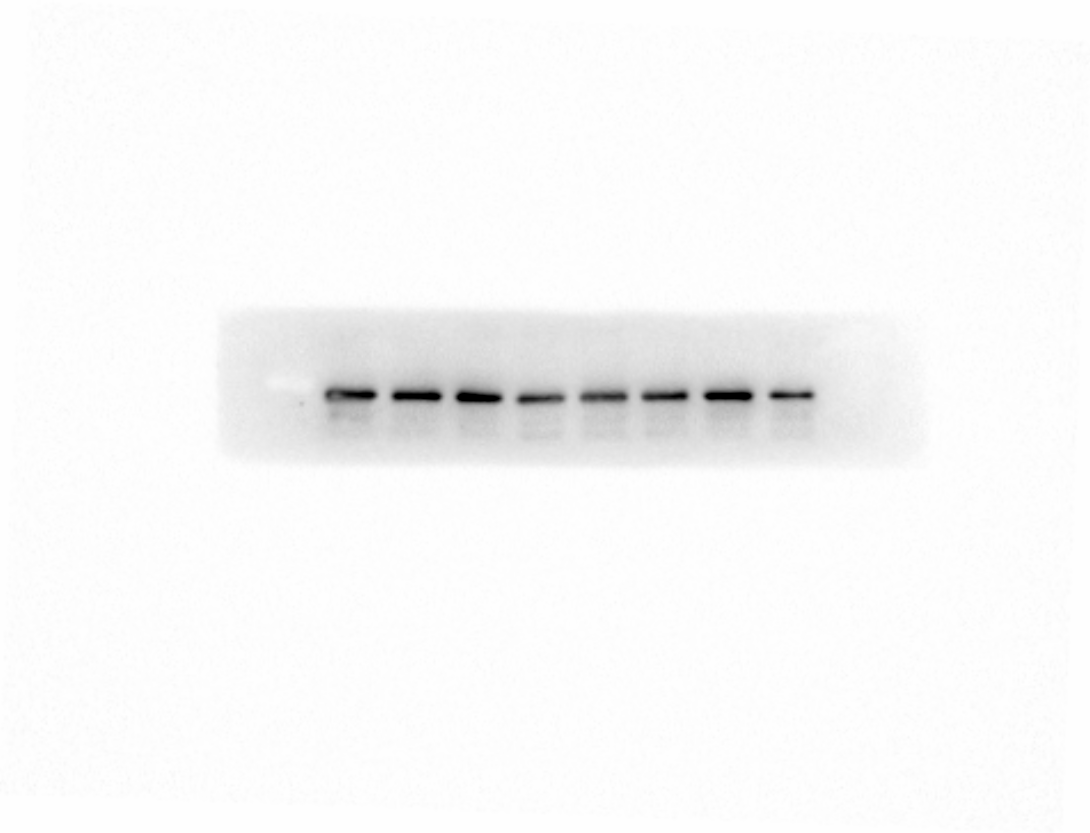

Supplement: Supplementary file 1 — Additional file 1.Figure S1-S15 Untreated GAPDH, p-JAK1, JAK1, p-STAT3 and STAT3 protein bands images (n=3). [file 12906_2020_2874_MOESM1_ESM.zip › 12906_2020_2874_MOESM1_ESM/Figure S10.tif]

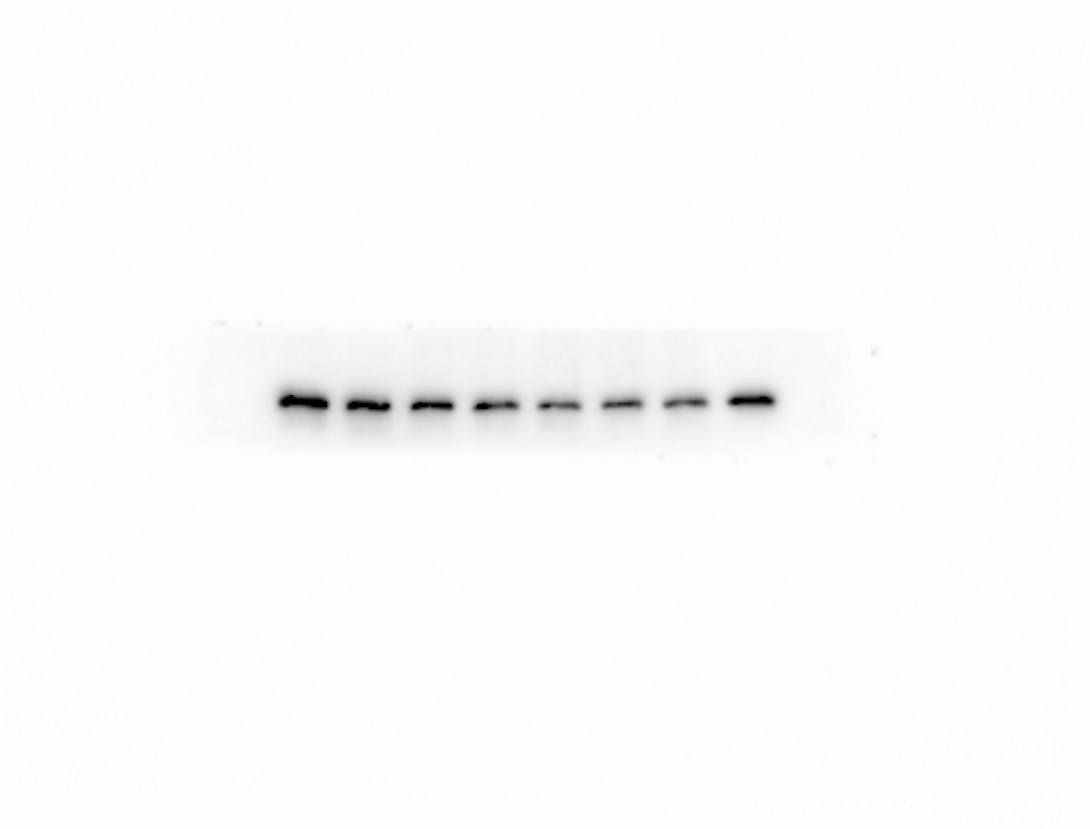

Supplement: Supplementary file 1 — Additional file 1.Figure S1-S15 Untreated GAPDH, p-JAK1, JAK1, p-STAT3 and STAT3 protein bands images (n=3). [file 12906_2020_2874_MOESM1_ESM.zip › 12906_2020_2874_MOESM1_ESM/Figure S11.tif]

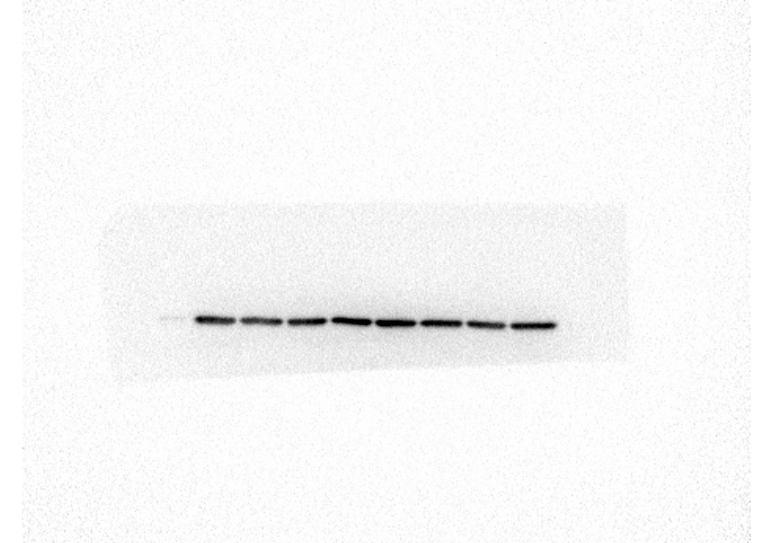

Supplement: Supplementary file 1 — Additional file 1.Figure S1-S15 Untreated GAPDH, p-JAK1, JAK1, p-STAT3 and STAT3 protein bands images (n=3). [file 12906_2020_2874_MOESM1_ESM.zip › 12906_2020_2874_MOESM1_ESM/Figure S12.tif]

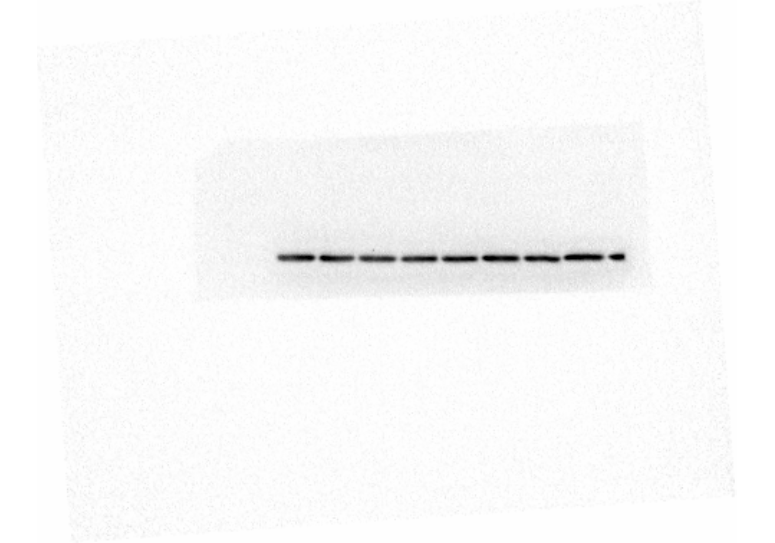

Supplement: Supplementary file 1 — Additional file 1.Figure S1-S15 Untreated GAPDH, p-JAK1, JAK1, p-STAT3 and STAT3 protein bands images (n=3). [file 12906_2020_2874_MOESM1_ESM.zip › 12906_2020_2874_MOESM1_ESM/Figure S13.tif]

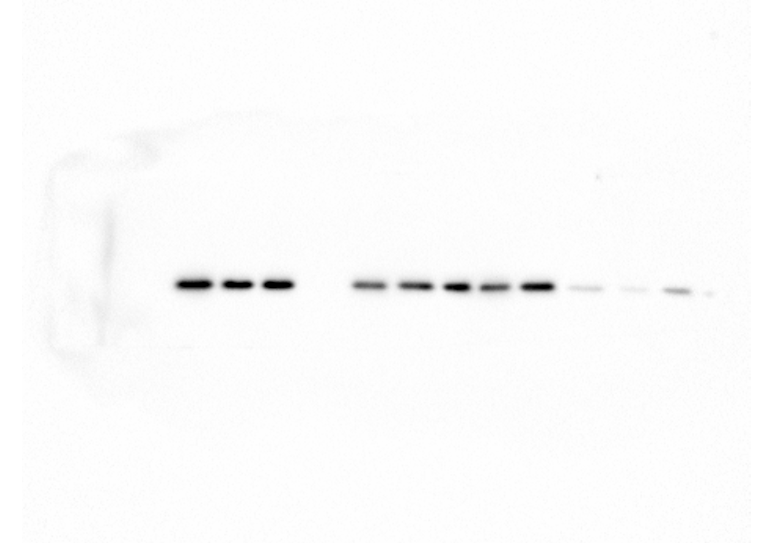

Supplement: Supplementary file 1 — Additional file 1.Figure S1-S15 Untreated GAPDH, p-JAK1, JAK1, p-STAT3 and STAT3 protein bands images (n=3). [file 12906_2020_2874_MOESM1_ESM.zip › 12906_2020_2874_MOESM1_ESM/Figure S14.tif]

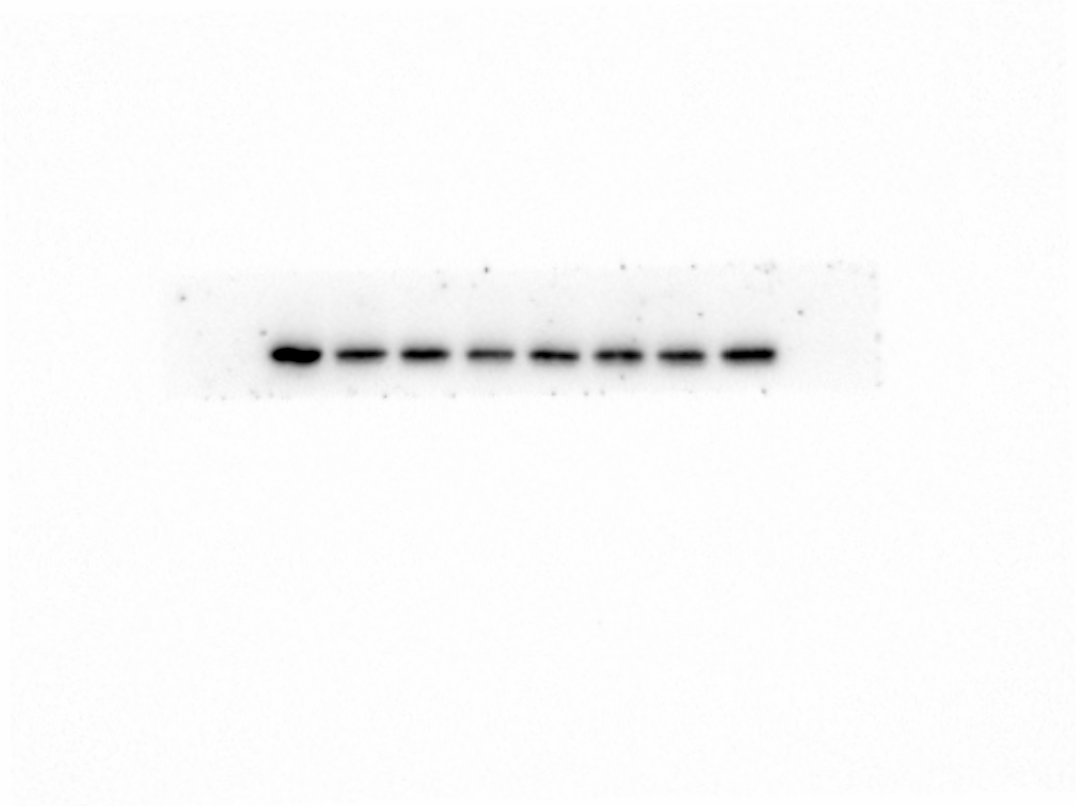

Supplement: Supplementary file 1 — Additional file 1.Figure S1-S15 Untreated GAPDH, p-JAK1, JAK1, p-STAT3 and STAT3 protein bands images (n=3). [file 12906_2020_2874_MOESM1_ESM.zip › 12906_2020_2874_MOESM1_ESM/Figure S15.tif]

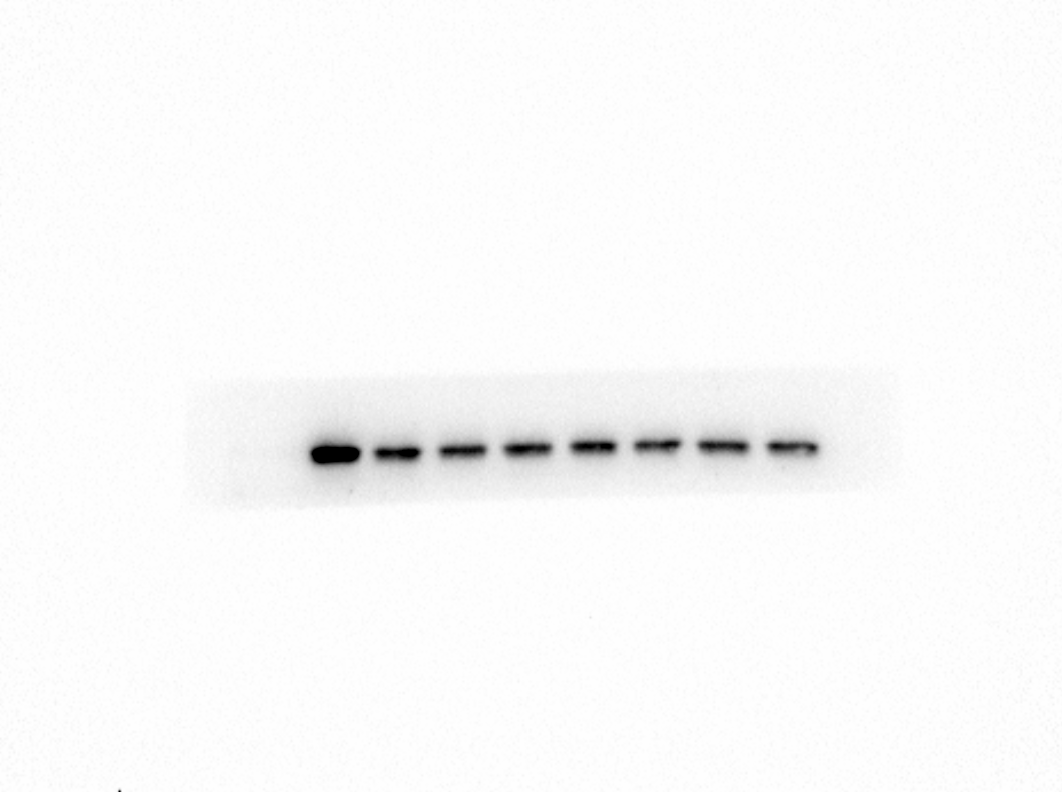

Supplement: Supplementary file 1 — Additional file 1.Figure S1-S15 Untreated GAPDH, p-JAK1, JAK1, p-STAT3 and STAT3 protein bands images (n=3). [file 12906_2020_2874_MOESM1_ESM.zip › 12906_2020_2874_MOESM1_ESM/Figure S2.tif]

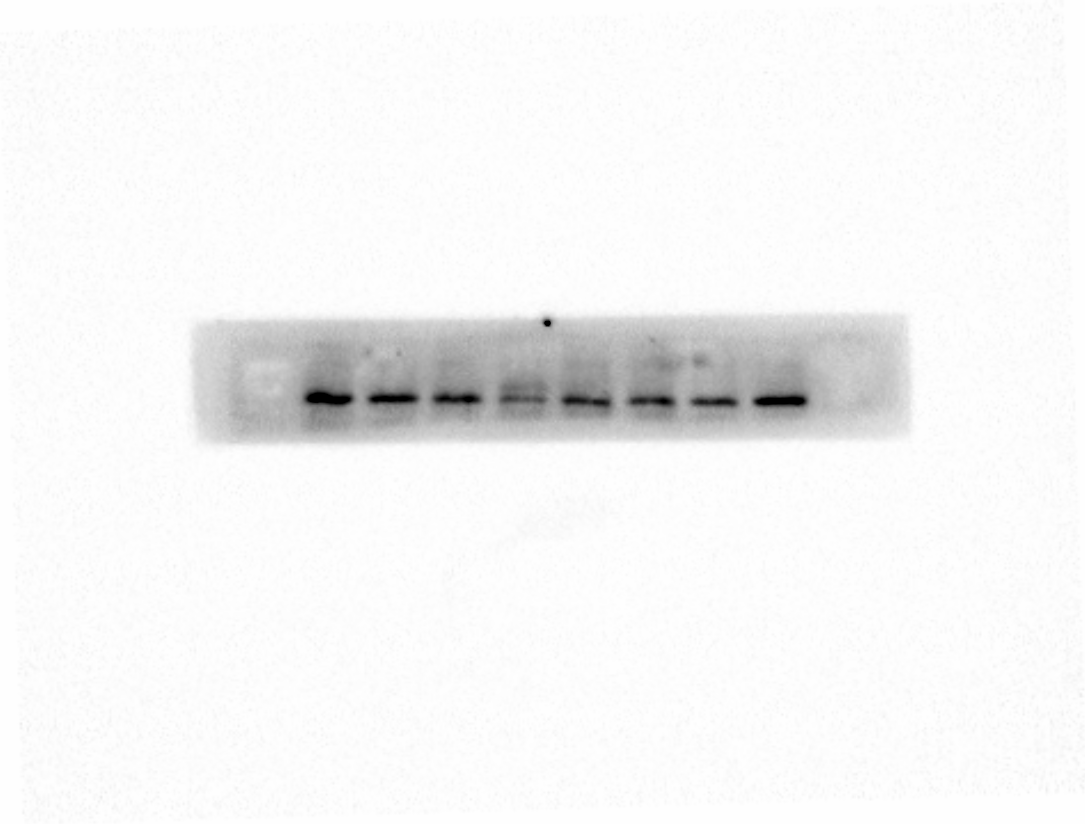

Supplement: Supplementary file 1 — Additional file 1.Figure S1-S15 Untreated GAPDH, p-JAK1, JAK1, p-STAT3 and STAT3 protein bands images (n=3). [file 12906_2020_2874_MOESM1_ESM.zip › 12906_2020_2874_MOESM1_ESM/Figure S3.tif]

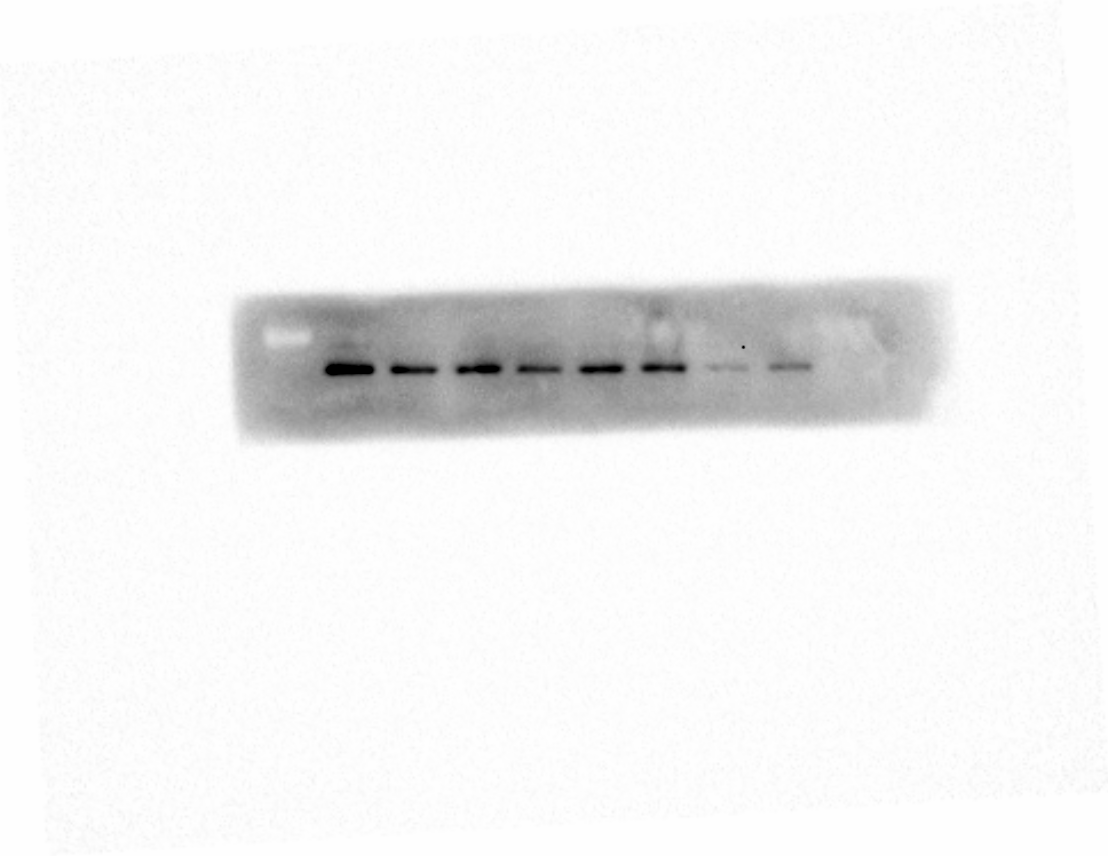

Supplement: Supplementary file 1 — Additional file 1.Figure S1-S15 Untreated GAPDH, p-JAK1, JAK1, p-STAT3 and STAT3 protein bands images (n=3). [file 12906_2020_2874_MOESM1_ESM.zip › 12906_2020_2874_MOESM1_ESM/Figure S4.tif]

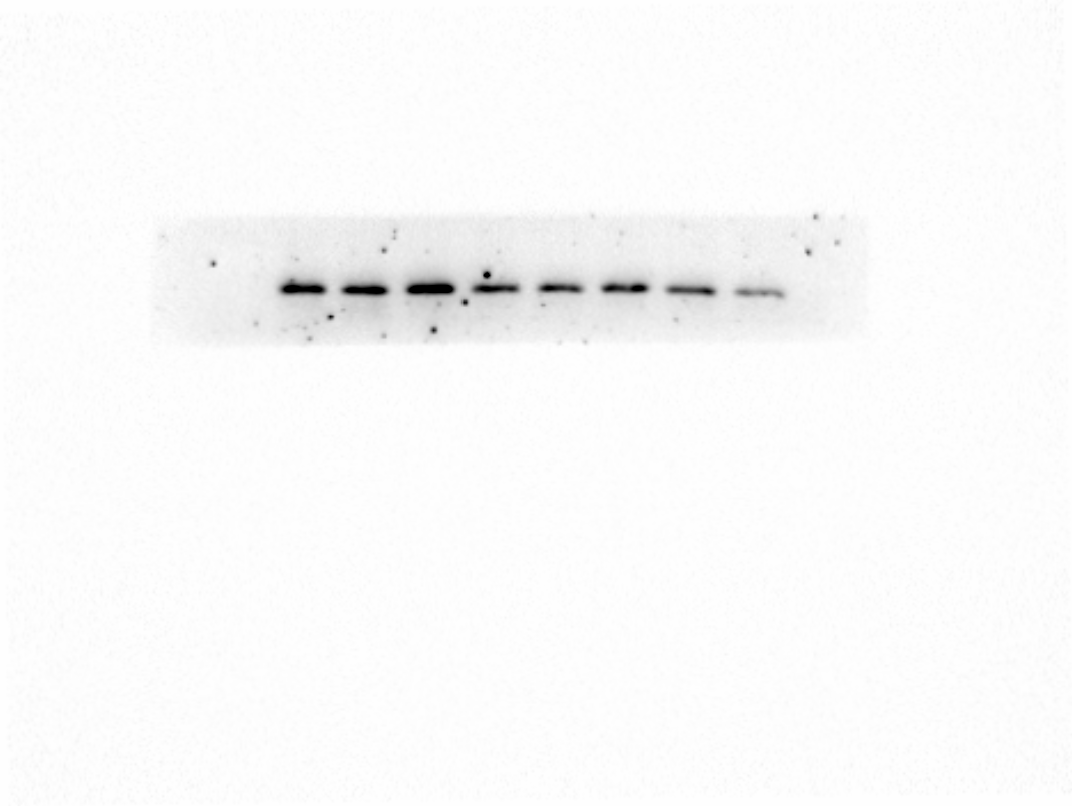

Supplement: Supplementary file 1 — Additional file 1.Figure S1-S15 Untreated GAPDH, p-JAK1, JAK1, p-STAT3 and STAT3 protein bands images (n=3). [file 12906_2020_2874_MOESM1_ESM.zip › 12906_2020_2874_MOESM1_ESM/Figure S5.tif]

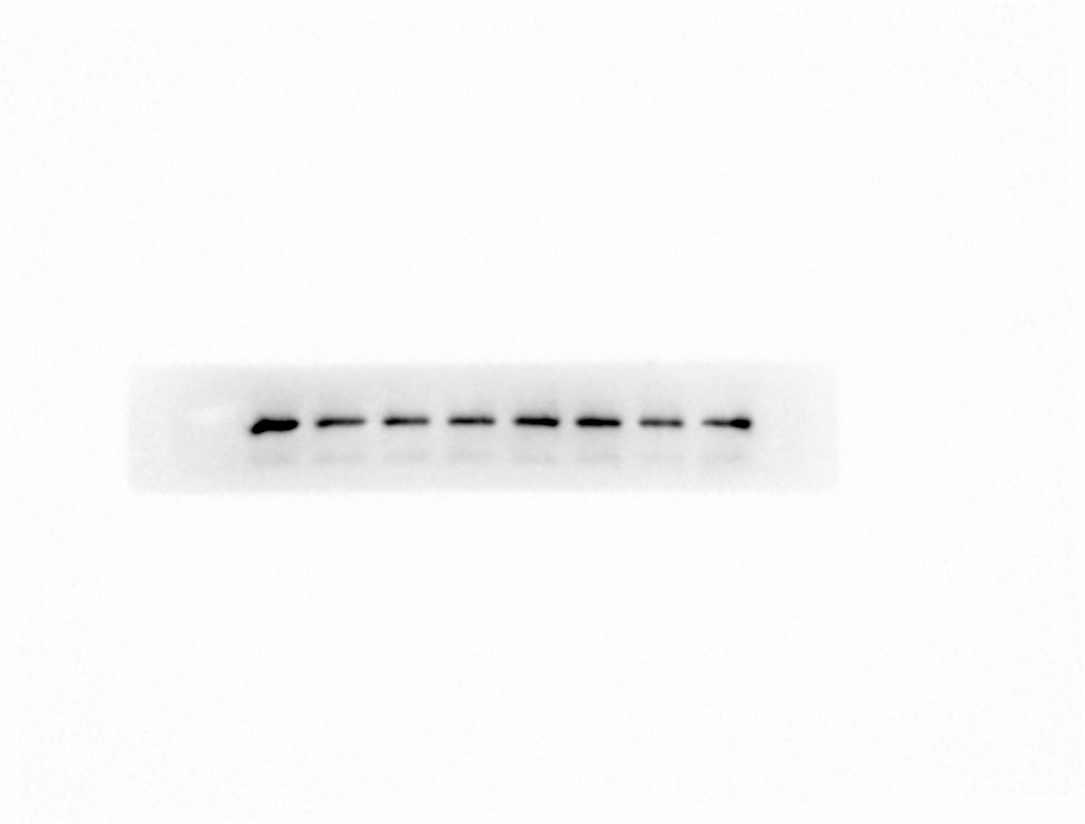

Supplement: Supplementary file 1 — Additional file 1.Figure S1-S15 Untreated GAPDH, p-JAK1, JAK1, p-STAT3 and STAT3 protein bands images (n=3). [file 12906_2020_2874_MOESM1_ESM.zip › 12906_2020_2874_MOESM1_ESM/Figure S6.tif]

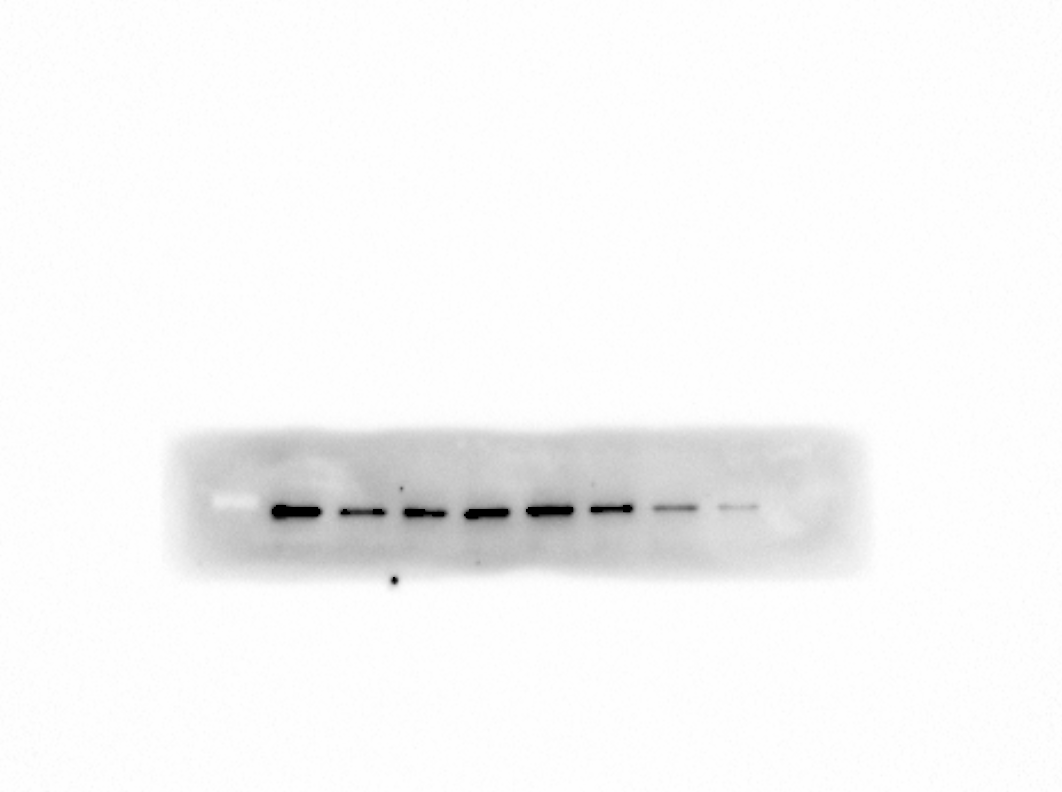

Supplement: Supplementary file 1 — Additional file 1.Figure S1-S15 Untreated GAPDH, p-JAK1, JAK1, p-STAT3 and STAT3 protein bands images (n=3). [file 12906_2020_2874_MOESM1_ESM.zip › 12906_2020_2874_MOESM1_ESM/Figure S7.tif]

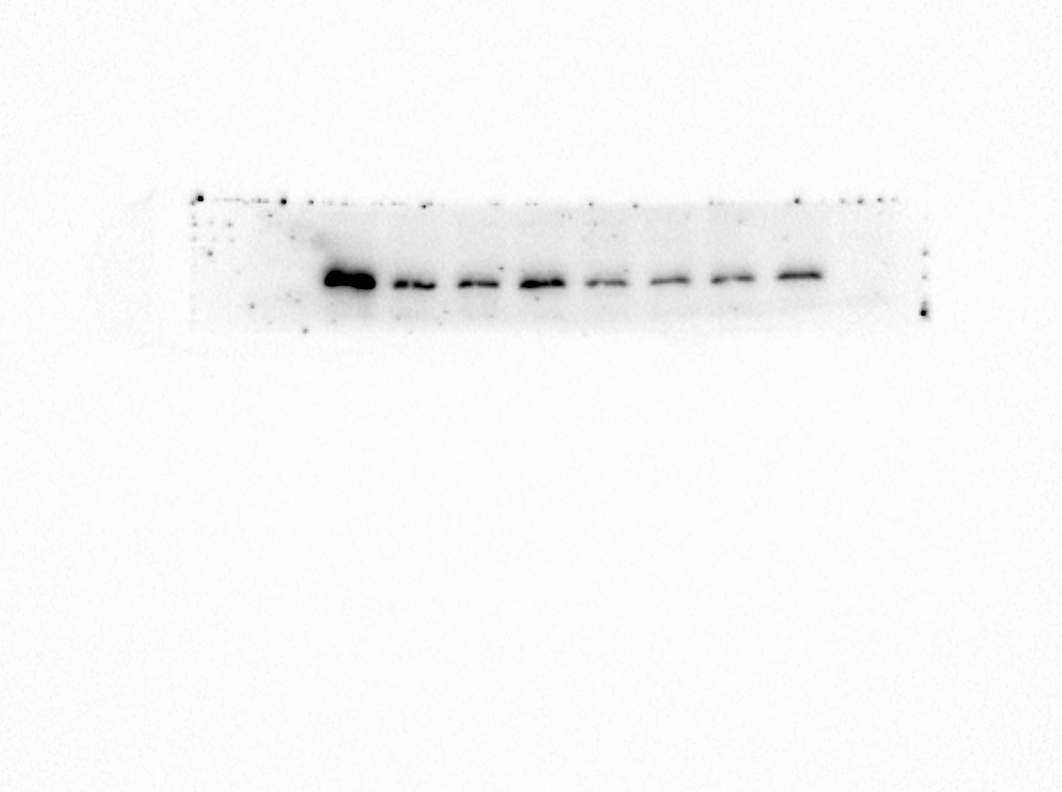

Supplement: Supplementary file 1 — Additional file 1.Figure S1-S15 Untreated GAPDH, p-JAK1, JAK1, p-STAT3 and STAT3 protein bands images (n=3). [file 12906_2020_2874_MOESM1_ESM.zip › 12906_2020_2874_MOESM1_ESM/Figure S8.tif]

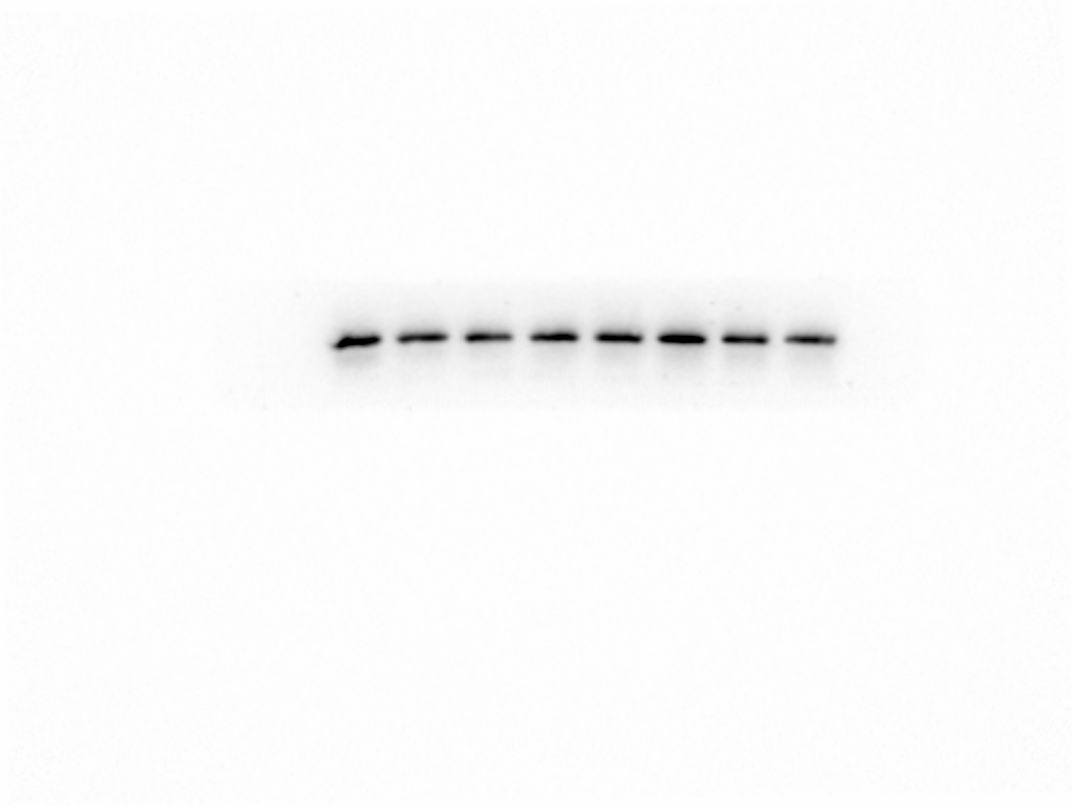

Supplement: Supplementary file 1 — Additional file 1.Figure S1-S15 Untreated GAPDH, p-JAK1, JAK1, p-STAT3 and STAT3 protein bands images (n=3). [file 12906_2020_2874_MOESM1_ESM.zip › 12906_2020_2874_MOESM1_ESM/Figure S9.tif]
